# CW-DETR: An Efficient Detection Transformer for Traffic Signs in Complex Weather

**DOI:** 10.3390/s26010325

**Published:** 2026-01-04

**Authors:** Tianpeng Wang, Qiaoshuang Teng, Shangyu Sun, Weidong Song, Jinhe Zhang, Yuxuan Li

**Affiliations:** 1 School of Geomatics, Liaoning Technical University, Fuxin 123000, China; 2 Collaborative Innovation Institute of Geospatial Information Service, Liaoning Technical University, Fuxin 123000, China; 3 State Key Laboratory of Information Engineering in Surveying, Mapping and Remote Sensing, Wuhan University, Wuhan 430079, China

**Keywords:** traffic sign detection, real-time detection, complex weather, multiscale feature fusion, edge enhancement, detection transformer

## Abstract

Traffic sign detection under adverse weather conditions remains challenging due to severe feature degradation caused by rain, fog, and snow, which significantly impairs the performance of existing detection systems. This study presents the CW-DETR (Complex Weather Detection Transformer), an end-to-end detection framework designed to address weather-induced feature deterioration in real-time applications. Building upon the RT-DETR, our approach integrates four key innovations: a multipath feature enhancement network (FPFENet) for preserving fine-grained textures, a Multiscale Edge Enhancement Module (MEEM) for combating boundary degradation, an adaptive dual-stream bidirectional feature pyramid network (ADBF-FPN) for cross-scale feature compensation, and a multiscale convolutional gating module (MCGM) for suppressing semantic–spatial confusion. Extensive experiments on the CCTSDB2021 dataset demonstrate that the CW-DETR achieves 69.0% AP and 94.4% $AP_{50}$, outperforming state-of-the-art real-time detectors by 2.3–5.7 percentage points while maintaining computational efficiency (56.8 GFLOPs). A cross-dataset evaluation on TT100K, the TSRD, CNTSSS, and real-world snow conditions (LNTU-TSD) confirms the robust generalization capabilities of the proposed model. These results establish CW-DETR as an effective solution for all-weather traffic sign detection in intelligent transportation systems.

## 1. Introduction

Traffic sign detection is a fundamental perception task in intelligent transportation systems [1]. Under adverse weather conditions such as fog, rain, and snow, the detection performance tends to degrade due to reduced contrast, dynamic noise patterns, and partial occlusion [2,3,4]. These factors affect the feature quality at multiple levels, from texture ambiguity to boundary blurring, presenting challenges for detection systems designed primarily for clear-weather scenarios.

Object detection has evolved through two-stage methods such as Faster R-CNN [5], single-stage approaches such as the YOLO series [6,7,8,9], and more recently, transformer-based frameworks such as the DETR [10] and RT-DETR [11]. While these architectures have achieved notable progress, their robustness under adverse weather conditions remains an active research direction [12,13].

To address the detection challenges posed by adverse weather conditions, various solutions have been proposed. DeMatchNet [14] achieves robust image matching through feature fusion and alignment modules. Ogino et al. [15] utilized Bézier curve-based filters to handle low-light environments, and Wang et al. [16] proposed a comprehensive framework that incorporates image preprocessing, an improved FPN architecture, and optimized loss functions. Qu et al. [17] employed coordinate attention mechanisms to counteract weather-induced interference. Chandnani et al. [18] adopted rainfall type classification to select appropriate classifiers, and Yacoob et al. [19] integrated QTNet and FCNet to mitigate the effects of rain and snow. These methods provide valuable insights, yet weather-induced degradation often varies across feature scales [20], suggesting room for scale-aware designs.

The detection of traffic signs can also refer to their inherent characteristics. Signs are often small relative to the image dimensions, and distinguishing texture details can diminish through successive downsampling [21,22]. Additionally, geometric boundaries carry category-relevant information, but conventional feature pyramids [23] may not explicitly preserve edge features under low-contrast conditions [24,25].

Building on these observations, we propose the CW-DETR (Complex Weather Detection Transformer), which is based on the RT-DETR. The contributions of this study include the following:

(1) We propose two complementary modules: the fused path feature enhancement module (FPFEM) preserves fine-grained texture features through multipath feature transformation and reparameterization, while the Multiscale Edge Enhancement Mechanism (MEEM) enhances boundary information via channel-decoupled edge extraction. Together, they address texture blurring and edge degradation under adverse weather conditions.
(2) We construct the Adaptive Dual-Branch Bidirectional Fusion Feature Pyramid Network (ADBF-FPN) for cross-scale feature compensation through bidirectional feature flow and adaptive weighted fusion, and design the multiscale convolutional gating module (MCGM) to selectively suppress weather-induced noise by using partial convolution and gated filtering. These modules jointly improve the multiscale detection robustness.
(3) On CCTSDB2021, the CW-DETR achieves 69.0% AP and 94.4% $AP_{50}$, outperforming the baseline by 5.7 and 4.5 percentage points respectively, while reducing parameters by 15%. Cross-dataset experiments on TT100K, the TSRD, CNTSSS, and real-world snow conditions validate the generalizability of the model.

The remainder of this paper is organized as follows: Section 2 reviews related work on object detection and the RT-DETR framework. Section 3 presents the proposed CW-DETR architecture, detailing the design of fused path feature enhancement Net (FPFENet), the MEEM, the ADBF-FPN, and the MCGM. Section 4 describes the experimental setup and presents comprehensive results including ablation studies, comparative experiments, and cross-dataset evaluations. Finally, Section 5 concludes the paper with a summary of findings and directions for future research.

## 2. Related Work

This section reviews object detection architectures relevant to our work, focusing on the evolution from CNN-based to transformer-based methods, and then details the RT-DETR framework that serves as our baseline.

### 2.1. Object Detection Algorithms

Object detection techniques are generally categorized into two-stage and single-stage detectors. Two-stage detectors such as the R-CNN [26] and Faster R-CNN [5] achieve high accuracy through region proposal networks but involve significant computational overhead. Single-stage detectors, pioneered by YOLO [6], reformulate detection as regression for real-time inference. The series has evolved through architectural improvements including feature pyramids, attention mechanisms, and efficient convolutions into YOLOv5-v12 [7,8,9]. In parallel, traffic sign recognition, the downstream task of classifying detected sign regions, has matured considerably, with spatial transformer networks achieving 99.71% accuracy on the German benchmark [27] and YOLOv5-based approaches reaching 97.70% mAP under normal conditions [28]. Nevertheless, comprehensive surveys indicate that both detection and recognition performance degrade substantially when faced with geographic variability, complex backgrounds, and adverse illumination [29], underscoring the need for weather-robust detection architectures.

Transformer-based detection has opened new avenues since the DETR [10] introduced end-to-end detection by eliminating anchors and the NMS introduced bipartite matching. However, the DETR suffers from slow convergence and suboptimal small object detection. The Deformable DETR [30] addresses this issue by replacing global attention with multiscale deformable attention. Subsequent works such as the Conditional DETR and DAB-DETR further refine the query mechanisms for faster convergence, and advanced feature fusion architectures such as the BiFPN [31], MAFPN [32], and MANet [33] have improved the detection robustness in low-visibility environments. These innovations have led to the RT-DETR [11], the first real-time end-to-end detector that outperforms the YOLO series in terms of both speed and accuracy.

### 2.2. RT-DETR Framework

The YOLO series currently dominates real-time detection applications. However, its speed and accuracy are constrained by the nonmaximum suppression (NMS) postprocessing step, which introduces latency in the detection pipeline [30]. With the emergence of transformer-based end-to-end detectors, such as the DETR [10], Zhao et al. [11] proposed a real-time detection transformer (RT-DETR), the first end-to-end detector capable of real-time performance. The RT-DETR eliminates the drawbacks of the NMS observed in YOLO models and can adapt to various scenarios without the need for retraining. The RT-DETR architecture consists of a backbone, an efficient hybrid encoder, and a transformer decoder with auxiliary prediction heads. Specifically, the RT-DETR inputs the features from the last three stages of the backbone network (S3, S4, and S5) into the encoder. The efficient hybrid encoder CNN-based cross-scale feature fusion (CCFF) performs intrascale feature interaction and cross-scale feature fusion to transform the multiscale features into a sequence of image features. Then, a minimum uncertainty query selection mechanism is used to select a fixed number of encoder features as the initial object queries for the decoder. Finally, the decoder, which is equipped with auxiliary prediction heads, iteratively refines these object queries to generate the final categories and bounding boxes.

Despite these advances, existing methods exhibit notable limitations regarding traffic sign detection under adverse weather conditions. First, the standard backbones progressively lose fine-grained textures during downsampling [22], a problem in which weather-induced blur is further exacerbated [34]. Second, the edge information critical for boundary localization degrades under low contrast, yet the current architectures generally lack explicit edge-preserving mechanisms [35]. Third, as noted by Li et al. [20], conventional multiscale fusion treats degradation uniformly across scales, ignoring the asymmetric nature of weather effects: shallow layers primarily lose spatial details while deep layers suffer semantic ambiguity. These observations motivate our CW-DETR framework, which addresses each gap via its four specialized modules.

## 3. Method Overview

**Figure 1:**
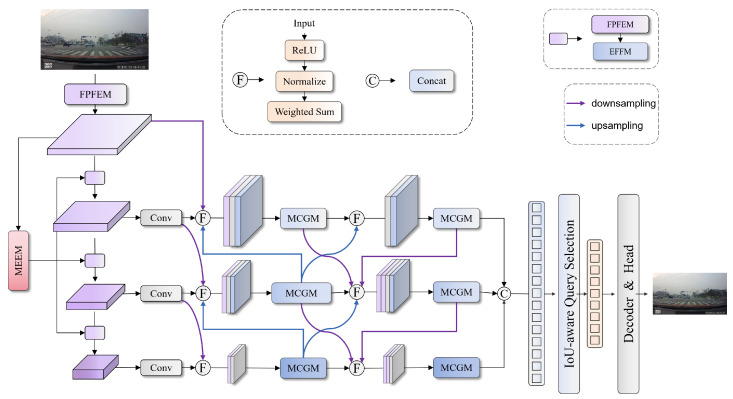
Overall architecture of the CW-DETR integrating FPFENet, the MEEM, the ADBF-FPN, and the MCGM for weather-robust traffic sign detection.

In this paper, we propose the CW-DETR model for traffic sign detection under complex weather conditions. Building upon the real-time end-to-end detection framework, the RT-DETR, the CW-DETR optimizes both feature extraction and feature fusion mechanisms. As illustrated in Figure 1, the input image is first processed by FPFENet to extract semantic features with preserved fine-grained textures. These features are subsequently enhanced by the MEEM to reinforce edge information and perform edge-semantic fusion. The multiscale features are further fused by the ADBF-FPN through bidirectional cross-scale information flow to restore degraded features. Finally, the MCGM filters the features to suppress weather-induced noise. The refined features are passed to the encoder for cross-scale interaction and decoded to produce detection results.

### 3.1. Multipath Feature Enhancement Backbone

**Figure 2:**
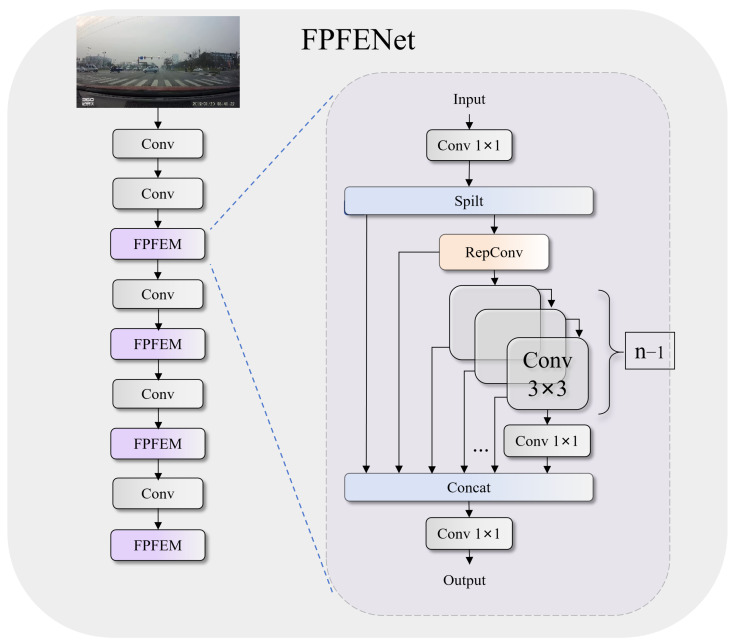
FPFENet backbone structure (**left**) and the FPFEM design with channel splitting and RepConv enhancement (**right**).

In complex traffic environments, traffic signs are often small and prone to being lost in feature maps [36]. To address this issue, we design FPFENet (see Figure 2), which employs multistage feature extraction and progressive feature aggregation strategies. By integrating a multibranch structure with reparameterization techniques, FPFENet enhances the model’s ability to detect small objects while maintaining the inference speed.

FPFENet begins by rapidly downsampling the input to extract basic texture features. In the subsequent stages, it leverages the fused path feature enhancement module (FPFEM) to fuse information across multiple scales, ensuring the stable feature representation of traffic signs under adverse weather conditions, such as rain and fog. The FPFEM applies parallel transformations along the channel dimension, enabling features from different receptive fields to interact. This approach significantly improves the model’s ability to recognize textures in low-contrast environments. The RepConv reparameterization structure, which uses multibranch heterogeneous convolutions during training to enrich feature expression, is introduced. During inference, these branches are fused into a single efficient convolution operation, ensuring minimal impact on speed.

The FPFEM (see Figure 2) splits the input traffic sign features across channels and fuses multiscale and multistage information, enhancing feature diversity and improving the recognition of sign textures at various scales. This design effectively mitigates blurring caused by fog, rain, and poor lighting conditions.

The main workflow is divided into three key stages: feature separation and projection, multipath feature transformation, and feature aggregation. In the feature separation and projection stage, the input features are first linearly projected into a latent space and then separated into two parallel branches, denoted by branch $Y_{\alpha }$ and branch $Y_{\beta }$. The first branch $Y_{\alpha }$ is retained directly as an identity mapping while the second branch $Y_{\beta }$ undergoes feature enhancement transformation to generate the initial features, which are then passed through cascaded nonlinear transformations to form multilevel feature representations as defined in Equations (1)–(3). Here, $Z_{i}$ denotes the feature representation at the *i*-th transformation stage: $Z_{0}$ represents the initial enhanced features after RepConv transformation, $Z_{1}$ through $Z_{n-1}$ are intermediate features from cascaded nonlinear transformations, and $Z_{n}$ is the final compressed output. $T_{\rho }$ is the RepConv transformation, $T_{\tau }^{i}$ denotes the *i*-th nonlinear transformation, and $T_{\gamma }$ performs channel compression.

(1) $$\begin{array}{cc} Z_{0} & =T_{\rho }\left(Y_{\beta }\right) \end{array}$$

(2) $$\begin{array}{cc} \;\;\;\;\;\;\;\;\;\;\;\;\;\;\;\;\;\;\;\;\;\;\;\;\;\;\;\;\;\;\;\;\;\;\;\;\;\;\;Z_{i} & =T_{\tau }^{i}\left(Z_{i-1}\right),\;i\in \left\{1,2,\ldots ,n-1\right\} \end{array}$$

(3) $$\begin{array}{cc} \;\;Z_{n} & =T_{\gamma }\left(Z_{n-1}\right) \end{array}$$

In the adaptive feature aggregation stage, all branches are concatenated along the channel dimension and then mapped to the final output γ.

Compared with the conventional BasicBlock, although the FPFEM introduces a multibranch structure, it effectively controls the increase in computational complexity through channel scaling and parameter sharing strategies. Specifically, given the input feature $X\in R^{B\times C_{i}\times H\times W}$ and output feature $\gamma \in R^{B\times C_{o}\times H\times W}$, the computational complexity of the FPFEM is as follows:

(4) $$O\left(BHWC_{i}C_{m}+BHW\left(n+2\right)C_{m}^{2}+BHWC_{m}\left(n+2\right)C_{\gamma }\right)$$

By introducing $C_{m}=\lambda C_{\gamma }$, where λ<1, the FPFEM achieves a better balance between computational efficiency and representational capacity than the conventional BasicBlock does.

**Figure 3:**
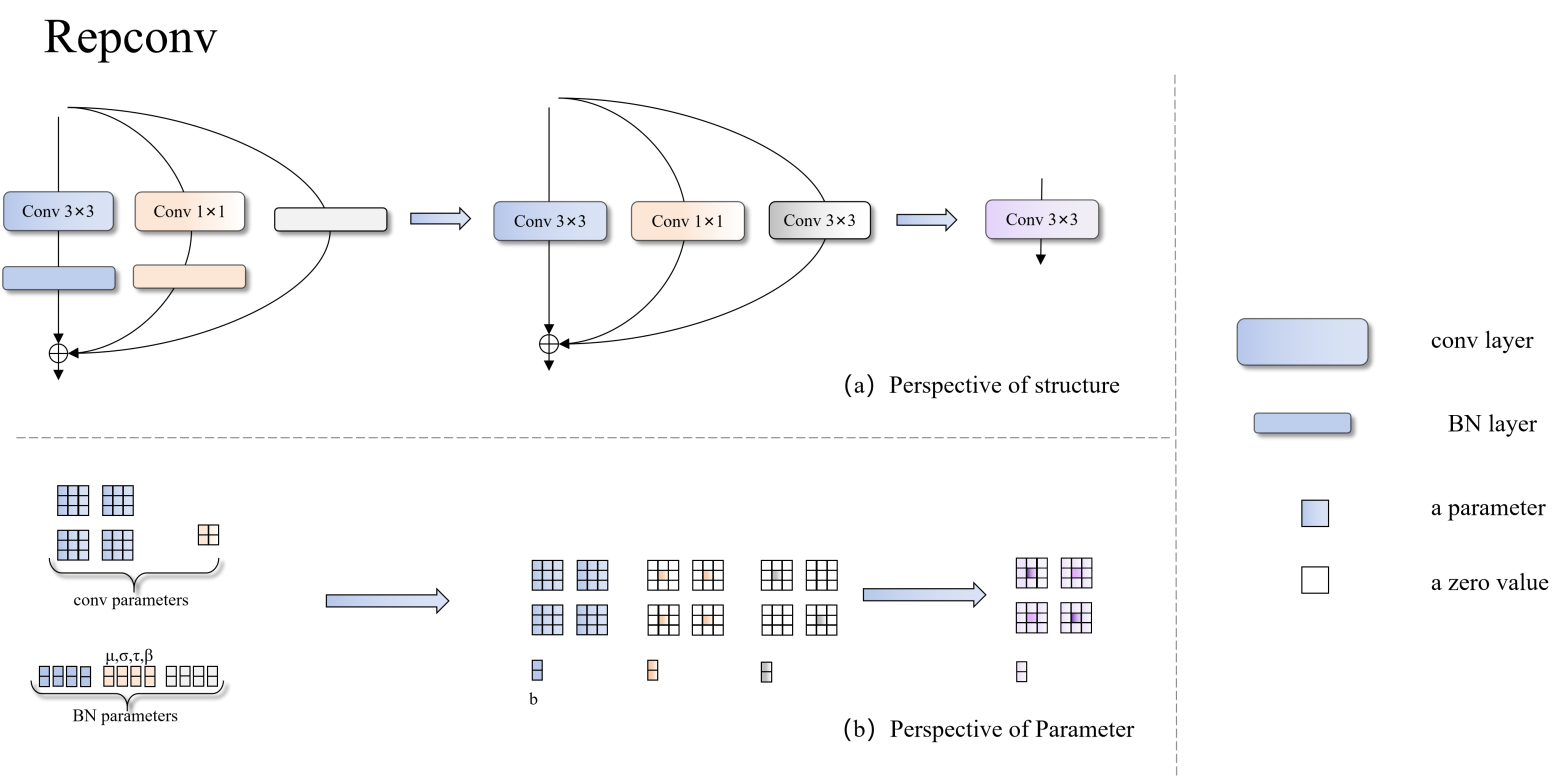
RepConv reparameterization: multibranch training (left) fused into a single convolution for inference.

RepConv is a model reparameterization technique that can merge multiple computational modules into one during the inference stage, thereby enhancing the model’s efficiency and performance [37]. RepConv consists of three parallel branches: a standard 3 × 3 convolution branch, a 1 × 1 pointwise convolution branch, and an identity mapping branch. These branches are designed to capture feature information from different receptive fields and spatial scales (Figure 3). During training, RepConv performs parallel forward computations through all three branches, each containing a convolutional layer followed by batch normalization. Prior to deployment, by leveraging the linearity of the convolution operations, the multibranch structure of RepConv is reparameterized into an equivalent single convolution, thus ensuring fast inference without compromising feature expressiveness.

Within the overall network architecture, FPFENet performs rapid downsampling followed by channelwise partitioning via the FPFEM to enhance feature reuse and reduce computational overhead. The integration of RepConv increases the feature diversity while preserving the inference speed, enabling robust fine-grained texture representation even when the input quality is degraded.

### 3.2. Multiscale Edge Enhancement Mechanism

While FPFENet enhances texture representation, the edge information that encodes geometric shape and positional cues requires separate treatment [38]. Standard feature extraction lacks explicit edge-preserving mechanisms, leading to boundary degradation under low-contrast conditions [39]. Thus, we propose the MEEM to address this gap through dedicated edge feature extraction and fusion.

The MEEM consists of two core components: (1) Multiscale edge information generator (MEIG): This module individually applies Sobel convolution across each channel to extract gradient information in both the horizontal and vertical directions. By isolating the edge computations to each channel, it effectively suppresses interchannel noise and produces high-fidelity initial edge maps. A bottom-up edge feature pyramid is subsequently constructed through successive max pooling and convolution operations, enabling the network to perceive the edge contours of traffic signs at multiple spatial scales. (2) Edge feature fusion module (EFFM): This module concatenates the fine-grained edge features generated by the MEIG with the semantic features from the backbone network along the channel dimension. Spatial convolution is then applied to fuse the information, followed by channel compression. This edge-semantic fusion strategy enhances the model’s ability to extract edge information under noisy conditions, such as rain and snow, thereby providing more robust features for subsequent detection tasks. In particular, for small-scale traffic signs, the enhanced multiscale edge features provide critical geometric cues, effectively alleviating the challenges of recognition under low-contrast conditions or occlusion.

#### 3.2.1. MEIG

**Figure 4:**
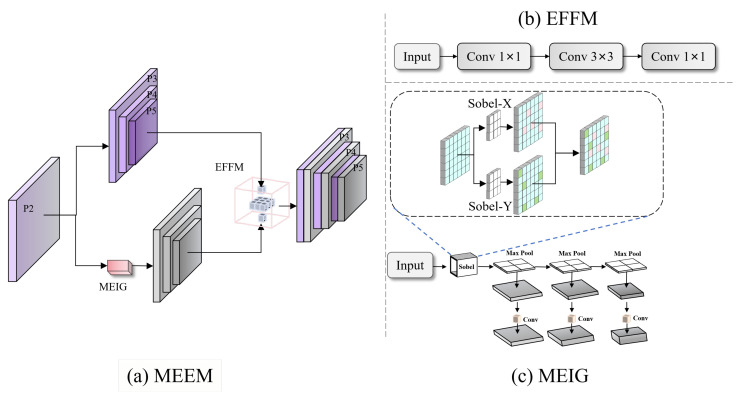
MEEM structure: (**a**) overall edge-semantic fusion; (**b**) EFFM fusion module; and (**c**) MEIG edge pyramid generator.

As illustrated in Figure 4c, the MEIG extracts edge information through channel-decoupled Sobel convolution and constructs a hierarchical edge feature pyramid.

**Channel-Decoupled Edge Extraction.** Unlike standard Sobel operators, which compute gradients on grayscale images, we apply Sobel filtering independently to each feature channel by using 3D grouped convolution. This design is motivated by two observations: (1) different channels encode different semantic patterns, and cross-channel gradient computation may introduce noise under adverse weather conditions, and (2) weather-induced artifacts (e.g., rain streaks and fog gradients) often exhibit channel-specific characteristics that independent processing can better isolate. The edge features are computed as follows:

(5) $$E_{G}\left(X\right)={\mathrm{Conv}3D}_{\mathrm{group}}^{H}\left(X\right)+{\mathrm{Conv}3D}_{\mathrm{group}}^{V}\left(X\right)$$

where ${\mathrm{Conv}3D}_{\mathrm{group}}^{H}$ and ${\mathrm{Conv}3D}_{\mathrm{group}}^{V}$ denote the horizontal and vertical Sobel filters implemented as grouped 3D convolutions with frozen weights, respectively, ensuring that each channel is processed independently.

**Edge Feature Pyramid.** The initial edge features are progressively downsampled through max pooling and channel-adjusted via 1×1 convolutions to construct a three-level pyramid $\left\{E_{S_{3}},E_{S_{4}},E_{S_{5}}\right\}$ aligned with the backbone features:

(6) $$E_{S_{i}}={\mathrm{Conv}}_{1\times 1}\left(\mathrm{MaxPool}\left(E_{S_{i-1}}\right)\right)$$

This pyramid enables the network to perceive edge contours at multiple spatial scales, which is critical for detecting traffic signs of varying sizes under low-contrast conditions.

#### 3.2.2. EFFM

To effectively address the challenge of fusing the edge and semantic features, we design the EFFMv (Figure 4b). This module first performs multiscale feature fusion along the channel dimension and then applies spatial convolution to extract fine-grained spatial structural information, thereby enhancing semantic representation while preserving edge details. Finally, a channel transformation is applied to project the fused features to the desired output dimensionality. The overall process can be formalized as follows:

(7) $$F_{F}=W_{C_{2}}\left(P_{S}\left(W_{C_{1}}\left(\left[F_{s},F_{e}\right]\right)\right)\right)$$

Let $F_{F}$ denote the fused output, [·,·] denote the channelwise concatenation operation, $F_{s}$ denote the semantic features from the backbone network, $F_{e}$ denote the edge features, $W_{C_{i}}$ denote the parameterized convolution layer, and $P_{S}$ denote the spatial perception operation.

Through its edge-semantic dual-channel structure, the MEEM complements FPFENet by providing explicit geometric cues that texture features alone cannot capture, enabling stable boundary localization even under degraded visibility.

### 3.3. Adaptive Dual-Branch Bidirectional Feature Pyramid Network

**Figure 5:**
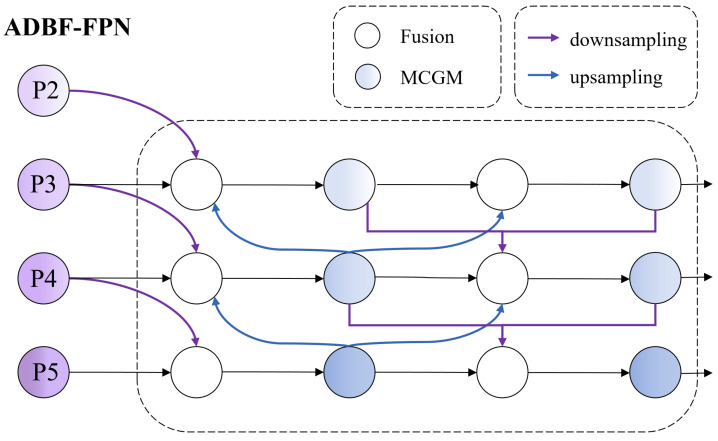
ADBF-FPN with bidirectional cross-scale feature flow and adaptive weighted fusion nodes.

Existing multiscale fusion methods such as the FPN [23] employ fixed top-down pathways, whereas the BiFPN [31] introduces bidirectional flows with learnable weights. However, the BiFPN applies uniform fusion across all scales without considering that weather-induced degradation affects different feature levels asymmetrically: shallow features primarily lose fine spatial details due to blur, whereas deep features suffer from semantic ambiguity caused by low contrast. To address this, we propose the ADBF-FPN (Figure 5) which has a dual-stream architecture in which an auxiliary path from P2 provides additional high-resolution information, and each fusion node learns independent weights to adaptively balance contributions based on scale-specific degradation patterns.

The ADBF-FPN consists of a main path and an auxiliary path. The main path incorporates a bidirectional feature transmission mechanism, including both top-down and bottom-up flows. In the top-down path, high-resolution shallow features are used to enhance the spatial localization ability of the deep semantic features. In the bottom-up path, semantic representations from deeper layers are propagated upward to compensate for the degradation of the shallow features affected by occlusions, such as rain or snow. The auxiliary path introduces additional information flows between the selected feature levels to reinforce the representational capability of the key layers. Through this bidirectional and multistream design, each feature level not only retains its own representational strength but also integrates complementary multiscale information from other levels. As a result, the ADBF-FPN achieves more expressive and robust feature representations in terms of both the feature depth and spatial scale, enabling more reliable detection of traffic signs under complex weather conditions.

Let the input at the *i*-th layer be denoted by $P_{i}^{\mathrm{in}}$. In the top-down pathway, an intermediate feature, $P_{i}^{\mathrm{td}}$, is generated, whereas in the bottom-up pathway, the updated output feature $P_{i}^{\mathrm{dt}}$ is obtained. The feature updating process integrates information from three sources: the previous stage of the current layer, the current stage of the adjacent layers, and earlier stages. This fusion process is formally defined in (8) and (9), where *w* represents the learnable weighting parameters and σ(·) denotes the activation function following a convolution or attention operation. This recurrent-like structure establishes an information flow mechanism similar to that of recurrent neural networks (RNNs), enabling the effective propagation and interaction of feature representations across different spatial scales.

(8) $$P_{i}^{\mathrm{td}}=\sigma \left(\frac{w_{1}\times P_{i}^{\mathrm{in}}+w_{2}\times \mathrm{Resize}\left(P_{i-1}^{\mathrm{in}}\right)+w_{3}\times \mathrm{Resize}\left(P_{i+1}^{\mathrm{in}}\right)}{w_{1}+w_{2}+w_{3}+\epsilon }\right)$$

(9) $$P_{i}^{\mathrm{dt}}=\sigma \left(\frac{w_{1}^{\prime }\times P_{i}^{\mathrm{in}}+w_{2}^{\prime }\times P_{i}^{\mathrm{td}}+w_{3}^{\prime }\times \mathrm{Resize}\left(P_{i+1}^{\mathrm{dt}}\right)}{w_{1}^{\prime }+w_{2}^{\prime }+w_{3}^{\prime }+\epsilon }\right)$$

#### Fusion Module

At each fusion node, we adopt the fast normalized fusion from BiFPN but extend it with learnable scale-specific weights. Given *n* input features ${\left\{X_{i}\right\}}_{i=1}^{n}$, the fusion is computed as follows:

(10) $$F=\sum_{i=1}^{n}w_{i}^{\prime }\cdot X_{i},\;\mathrm{where}\;w_{i}^{\prime }=\frac{\mathrm{ReLU}(w_{i})}{\sum_{j=1}^{n}\mathrm{ReLU}\left(w_{j}\right)+\epsilon }$$

where $w_{i}$ represents the learnable parameters, which are initialized to 1, and $\epsilon =10^{-4}$ ensures numerical stability. Unlike BiFPN’s uniform fusion, each fusion node in the ADBF-FPN learns independent weights, allowing the network to adaptively balance contributions from different scales based on the weather-specific degradation patterns.

Through multipath feature flow, the ADBF-FPN enables each feature level to receive contextual information from multiple scales, facilitating comprehensive interaction in both the depth and scale dimensions while maintaining computational efficiency.

### 3.4. Multiscale Gated Filtering Mechanism

During multiscale fusion, features from different scales may contain inconsistent semantic information, leading to localization. This problem is exacerbated under adverse weather conditions in which noise patterns vary across scales. While channel attention mechanisms such as SE-Net [40] address this issue via global channel recalibration, they lack the spatial selectivity needed to filter location-dependent weather artifacts. Recent efficient designs such as the partial convolution in FasterNet [41] reduce computation but do not provide gating for selective suppression. Our MCGM combines both advantages: it uses partial convolution to process only 1/4 of the channels for efficiency, whereas a convolutional gated linear unit (ConvGLU) provides spatially aware filtering to suppress location-specific interference such as rain streaks and fog gradients.

**Figure 6:**
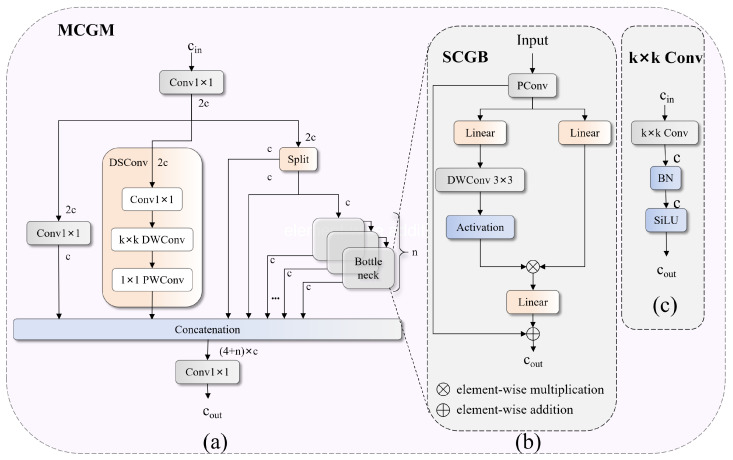
MCGM structure: (**a**) multibranch architecture; (**b**) SCGB with gated filtering; and (**c**) convolution block.

As illustrated in Figure 6a, the MCGM adopts a hierarchical multibranch architecture. Given an input feature tensor $X\in R^{C_{in}\times H\times W}$, the module first projects it to a higher-dimensional space through a 1×1 convolution, yielding $Y\in R^{2C\times H\times W}$, where *C* is the hidden channel dimension. The projected features are then processed through four parallel branches: (1) a linear compression branch that employs 1×1 convolution to preserve the global semantics; (2) a spatial enhancement branch with depthwise convolution that extracts local patterns; and (3–4) two identity branches obtained via channelwise splitting to retain the original information. The features from the fourth branch are further refined through *n* cascaded Spatial Contextual Gating Blocks (SCGBs). Finally, all of the branches are concatenated and compressed through a 1×1 convolution to produce the output $Z\in R^{C_{out}\times H\times W}$.

The SCGB, shown in Figure 6b, serves as the core filtering unit of the MCGM. It integrates partial convolution with a convolutional gated linear unit (ConvGLU) to achieve efficient spatial-aware feature modulation. Given an input $X_{in}$, the forward process is as follows:

(11) $$X_{out}=X_{in}+\mathrm{DropPath}\left(\mathrm{ConvGLU}\left(\mathrm{PConv}(X_{in})\right)\right)$$

The partial convolution (PConv) applies a 3×3 convolution to only 1/4 of the input channels while keeping the remaining 3/4 unchanged. This design reduces the computational cost by approximately 75% and maintains direct gradient paths through the unchanged channels for stable training. DropPath denotes stochastic depth regularization with drop rate p=0.1, and the ConvGLU, illustrated in Figure 6c, implements a spatially aware gating mechanism. Given an input $X\in R^{C\times H\times W}$, a 1×1 convolution first projects it to 2h channels (where h=2C/3), which are then split into gating branch *G* and value branch *V*. The gating branch passes through a 3×3 depthwise convolution and GELU activation to generate position-aware gating signals. The complete operation with residual connection is as follows:

(12) $$X_{out}=X+{\mathrm{Conv}}_{1\times 1}\left(\sigma \left({\mathrm{DWConv}}_{3\times 3}\left(G\right)\right)⊙V\right)$$

where σ(·) denotes GELU activation and ⊙ indicates elementwise multiplication. Depthwise convolution serves as position encoding, enabling spatially varying gating responses that adapt to location-dependent weather noise patterns such as rain streaks and fog gradients.

The MCGM incorporates several design choices to ensure stable training: (1) dual residual connections in both the SCGB and ConvGLU provide direct gradient paths, preventing vanishing gradients even when the gating values approach zero; (2) partial convolution processes only 1/4 of the channels, reducing the effective depth while the unchanged 3/4 of the channels serve as gradient shortcuts; (3) DropPath regularization (p=0.1) prevents overfitting to specific weather patterns; and (4) reduced hidden dimension (h=2C/3) in ConvGLU controls the parameter count and mitigates gradient explosion.

## 4. Experiments

### 4.1. Dataset

**Figure 7:**
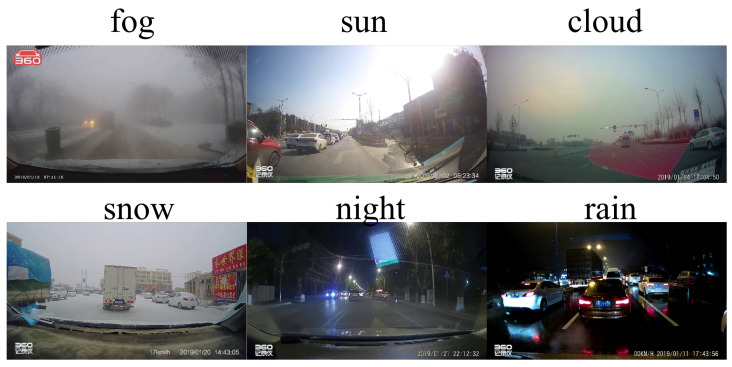
CCTSDB2021 samples under six weather conditions: fog, sun, cloud, snow, night, and rain.

The CCTSDB2021 dataset [37,38,39,42,43], which was developed by scholars and research teams from the Changsha University of Science and Technology, was collected from normal traffic scenes on roads in China. It contains approximately 20,000 images of traffic sign samples, comprising nearly 40,000 individual traffic signs. The dataset is divided into a training set, a testing set, a classification test set, and negative samples, and the annotations are categorized into three main types: prohibitory, mandatory, and warning. Unlike the official CCTSDB2021 partition designed for general traffic sign detection, our work specifically targets weather-robust detection. Therefore, we selectively utilize the classification test set, which uniquely provides images under six distinct weather and lighting conditions: cloud, fog, night, rain, snow, and sun (Figure 7), totaling 1500 images. Combined with 500 negative samples and supplementary data, we construct a weather-focused subset of 2000 images, which is divided into training, validation, and test sets at a 7:2:1 ratio. This deliberate selection ensures that the model is trained and evaluated specifically on weather-diverse scenarios, enabling fair assessment of the weather-adaptive detection capabilities rather than general detection performance.

### 4.2. Experimental Details

#### 4.2.1. Experimental Environment

All the experiments in this study were conducted using Python 3.8.18 and the deep learning framework consisted of PyTorch 1.13.1+cu117. The experiments were run on a single GPU, an NVIDIA GeForce RTX 3090 with 24 GB of VRAM, with CUDA 12.4 to fully leverage GPU acceleration. All of the input images were resized to 640 × 640 pixels to match the model’s input requirements.

The model was trained for 300 epochs with a batch size of eight to ensure training stability and efficiency. During training, the standard learning rate scheduling and optimizer configurations were adopted to enable the model to achieve high accuracy given limited computational resources and time.

#### 4.2.2. Evaluation Metrics

To comprehensively evaluate the performance of the CW-DETR, we adopted the COCO evaluation protocol with metrics for both detection accuracy and computational efficiency.

Detection Accuracy Metrics: The core metric is the average precision (AP), which is computed as the area under the precision–recall curve:

(13) $$\mathrm{AP}=\int_{0}^{1}p\left(r\right)\;dr$$

where p(r) denotes the precision at the recall level *r*. We report the AP averaged over IoU thresholds from 0.50 to 0.75 with 0.05 increments. Additionally, ${\mathrm{AP}}_{50}$ and ${\mathrm{AP}}_{75}$ are used to evaluate the performance at specific IoU thresholds:

(14) $${\mathrm{AP}}_{\tau }=\frac{1}{\left|C\right|}\sum_{c\in C}{\mathrm{AP}}_{c}^{\tau }$$

where τ∈{0.50,0.75} is the IoU threshold and *C* is the set of categories. Scale-specific metrics including ${\mathrm{AP}}_{S}$, ${\mathrm{AP}}_{M}$, and ${\mathrm{AP}}_{L}$ are used to assess the detection performance to evaluate the small, medium, and large objects, respectively.

Computational Efficiency Metrics: We measure the GFLOPs (giga floating-point operations) to evaluate the computational complexity and the parameter count as it relates to the model size. The inference speed is evaluated by using frames per second (FPS):

(15) $$\mathrm{FPS}=\frac{N}{T_{\mathrm{total}}}$$

where *N* is the number of test images and $T_{\mathrm{total}}$ is the total inference time excluding data loading and postprocessing. All speed measurements are conducted on a single NVIDIA RTX 3090 GPU with a batch size of two and an input resolution of 640×640.

Classification Metrics: We analyze false positive suppression by using column-normalized confusion matrices, where each column represents a true class and sums to 1. The notation BG→i denotes the proportion of true background samples predicted as class *i*. The per-class accuracy ${\mathrm{Acc}}_{i}$ is the diagonal element for class *i*, and the mean class accuracy is $\mathrm{MCA}=\frac{1}{\left|C\right|}\sum_{i\in C}{\mathrm{Acc}}_{i}$.

### 4.3. Ablation Study

Table 1 summarizes the ablation study results, where we progressively integrated FPFENet (A), the MEEM (B), the ADBF-FPN (C), and the MCGM (D) into the baseline RT-DETR-R18. The full CW-DETR architecture achieves 69.0% AP and 94.4% ${\mathrm{AP}}_{50}$, representing improvements of 5.7 and 4.5 percentage points, respectively, over the baseline, while the parameter count is reduced from 19.88 M to 16.88 M. This reduction is primarily attributed to FPFENet’s reparameterization design, which consolidates multibranch training representations into a single efficient convolution during inference. The MCGM further contributes to the computational efficiency: as shown in Table 1, adding the MCGM alone (+D) reduces the number of GFLOPs from 56.9 to 51.4, a 9.7% reduction. This efficiency gain stems from its overall architectural design that replaces the original RepC3 modules with a more efficient structure, where partial convolution processes only one quarter of input channels while the remaining three quarters pass through unchanged, and the ConvGLU employs a reduced hidden dimension of 2C/3. This design simultaneously reduces computation and provides gradient shortcuts for stable training.

Table 2 presents the background false positive distribution from the confusion matrices. FPFENet reduces BG→0 (mandatory sign false positives) from 0.38 to 0.17, a 55% reduction, by providing discriminative texture features. The MEEM alone introduces BG→2 false positives (0.18) due to edge hallucination, but this phenomenon is suppressed to 0.03 in the full model where the MCGM filters spurious edge responses. Notably, the MCGM alone exhibits degraded accuracy (${\mathrm{Acc}}_{2}$: 0.77), confirming that effective gating requires enriched upstream features. The full CW-DETR achieves 95.0% MCA with minimal class 2 false positives (0.03), demonstrating synergistic suppression across all modules.

When examining the contributions of individual modules, FPFENet achieves a 2.2 improvement in the AP while reducing the number of parameters by 30% and the number of GFLOPs by 22%, confirming that the multipath feature transformation preserves the texture information critical for small sign recognition without increasing the inference cost. The MEEM improves the $AP_{50}$ from 89.9% to 92.1%, indicating that edge enhancement strengthens boundary features and reduces missed detections at moderate localization thresholds. The overall AP decreases because the Sobel-based edge extraction amplifies the high-frequency responses, which benefits boundary-aware detection but can affect bounding box regression precision at stricter IoU thresholds—${\mathrm{AP}}_{75}$ changes from 76.6% to 72.8%. This behavior is expected for edge-centric modules operating without semantic context, and the effect is mitigated when MEEM is combined with FPFENet: the combination (+A+B) reaches 92.2% ${\mathrm{AP}}_{50}$ while recovering the overall AP to 64.8%, as the texture features from FPFENet provide semantic grounding which stabilizes the edge representations.

**Figure 8:**
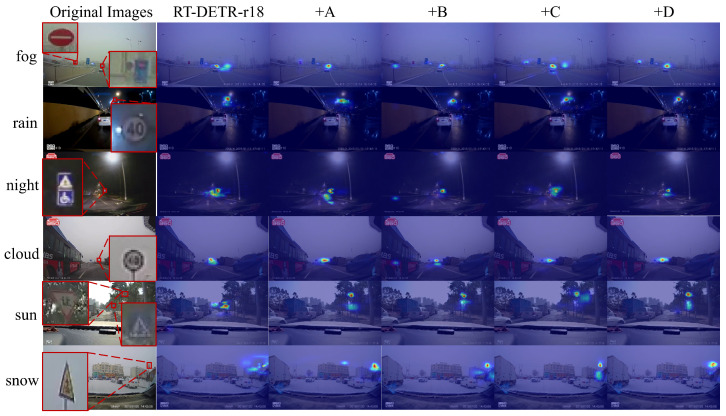
Ablation study visualization: Grad-CAM comparison showing progressive module contributions across weather conditions. The heatmap colors range from blue (low activation) to red (high activation), indicating the intensity of model attention.

The Grad-CAM visualization in Figure 8 reveals how each module affects detection behavior across weather conditions. In foggy scenes, the baseline produces diffuse activation spread across low-contrast regions, leading to potential false positives on background structures. Adding FPFENet concentrates the activation toward target regions by enhancing the discriminative texture features, effectively suppressing the responses to homogeneous foggy backgrounds. Furthermore, rainy-condition scenarios demonstrate the contribution of the MEEM to boundary discrimination: the sign contour becomes more distinctly separated from the rain-induced noise, reducing the confusion between the target edge and streak patterns. In night scenes, the baseline and early module combinations exhibit secondary activations on streetlights and reflective roads, which are sources of false positives under low-light conditions. The addition of the MCGM in the final column visibly suppresses these nontarget responses while preserving strong activation on the actual traffic sign; this selective filtering is the direct result of the gating mechanism learning to distinguish target-relevant features from scene-specific interference. The snow scenario has a cumulative effect: FPFENet maintains texture despite contrast reduction, the MEEM delineates the warning sign’s triangular boundary from the snow-covered background, and the MCGM filters the residual false activations on bright snow patches that share similar intensity patterns with the sign.

The transition from +A+B+C to the full model warrants attention from a quantitative perspective. The MCGM contributes a 4.0 percentage point AP improvement in this configuration, compared with 1.5 points when added to the baseline alone. This amplified contribution occurs because the MCGM’s gating operates more effectively on the enriched feature representations produced by the upstream modules: FPFENet provides diverse texture patterns, the MEEM supplies boundary cues, and the ADBF-FPN ensures cross-scale consistency. The gating mechanism can then make more informed decisions about which features to preserve and which to suppress. The full architecture achieves 68.3% ${\mathrm{AP}}_{S}$, a 6.5 percentage point improvement over the baseline’s 61.8%, demonstrating that this combination of modules particularly benefits small object detection when both texture preservation and false positive suppression are critical.

### 4.4. Comparative Experiments

**Figure 9:**
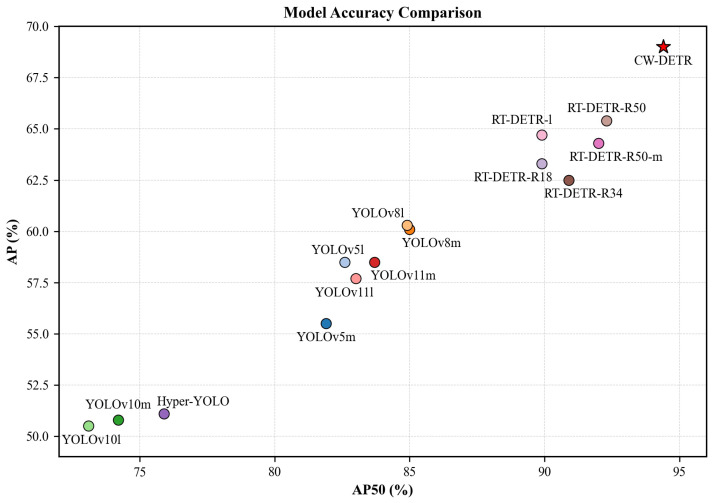
$APvsAP_{50}$ trade-off comparison. The CW-DETR achieves optimal accuracy–efficiency balance.

To comprehensively validate the effectiveness of the CW-DETR for traffic sign detection under complex weather conditions, we constructed an evaluation framework that includes three categories of representative models: mainstream real-time detectors, end-to-end detectors, and real-time end-to-end detectors. The experimental results are shown in Table 3 and Figure 9.

**Accuracy–Efficiency Trade-Off.** Among all of the compared methods, the CW-DETR achieves the best accuracy–efficiency balance. The CW-DETR attains 69.0% AP and 94.4% ${\mathrm{AP}}_{50}$, outperforming the RT-DETR-R18 by 5.7 and 4.5 percentage points respectively, while achieving 4.8% faster inference with 15% fewer parameters. Compared with larger models such as the RT-DETR-R50, the CW-DETR delivers higher accuracy at 85% faster speed. Although YOLO variants such as YOLOv10m achieve higher FPS, their accuracy falls substantially short, particularly for small objects.

**Figure 10:**
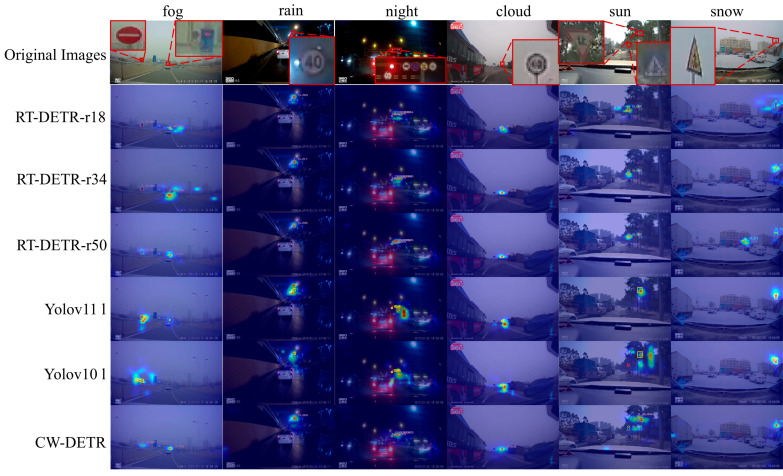
Comparative experiment visualization: Grad-CAM comparison between CW-DETR and state-of-the-art detectors across weather conditions. The heatmap colors range from blue (low activation) to red (high activation), indicating the intensity of model attention.

**Cross-Weather Robustness.** Figure 10 presents Grad-CAM visualizations across six weather conditions. Under fog and rain, the baseline methods produce diffuse attention patterns that fail to localize signs precisely, whereas the CW-DETR generates focused activation on the target regions. In night scenes, the CW-DETR maintains strong responses to traffic signs despite low contrast, whereas the other methods exhibit scattered attention. Under snow conditions in which signs are partially occluded, the CW-DETR still produces accurate localization. These visualizations confirm that the CW-DETR’s systematic design, combining edge enhancement and gated filtering, effectively suppresses weather-induced interference while preserving discriminative features.

**Figure 11:**
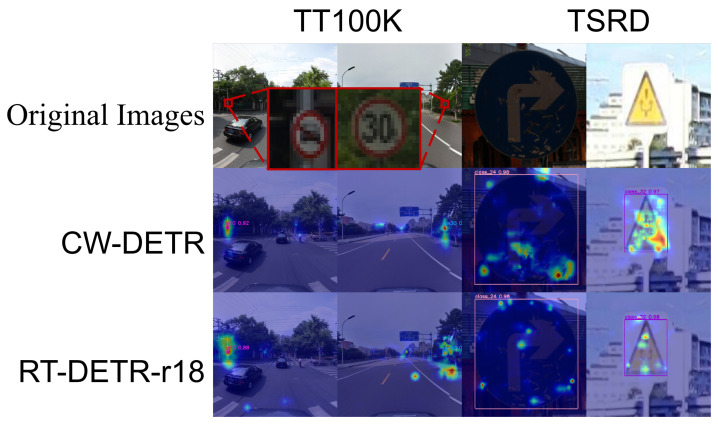
Transfer learning visualization: Grad-CAM comparison on TT100K and TSRD datasets. The heatmap colors range from blue (low activation) to red (high activation), indicating the intensity of model attention.

To comprehensively evaluate the generalization capability of the CW-DETR, we conduct experiments on four additional datasets: the TT100K [44], the TSRD for supervised transfer learning, and the LNTU-TSD and CNTSSS [45] for zero-shot evaluation under adverse weather conditions. Table 4 presents the transfer learning results on the TT100K and TSRD datasets. On TT100K, which contains small and densely distributed traffic signs in complex urban scenes, the CW-DETR achieves 67.7% AP and 83.0% ${AP}_{50}$, outperforming the RT-DETR-R18 by 3.9 and 4.5 percentage points, respectively. The improvement is particularly notable for small objects, demonstrating that FPFENet’s texture preservation benefits cross-domain small sign detection. With respect to the TSRD, on which the baseline models already achieve high accuracy, the CW-DETR further improves the ${AP}_{L}$ to 89.0%. Figure 11 shows that, compared with the RT-DETR-R18, the CW-DETR produces more focused attention patterns on target regions which exhibit scattered activation in background areas.

**Figure 12:**
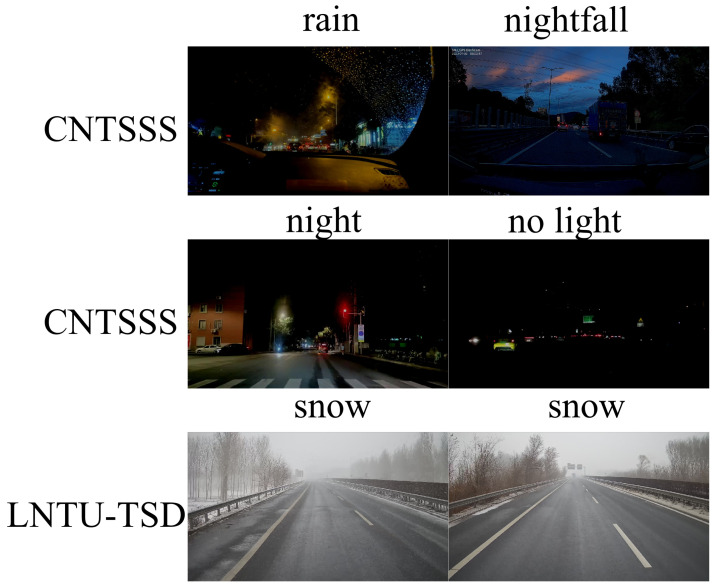
Zero-shot test datasets: CNTSSS (night conditions) and LNTU-TSD (real snow scenes).

To comprehensively evaluate the generalization ability of the CW-DETR, we conducted experiments on two zero-shot datasets. The CNTSSS dataset comprises four low-light scenarios: nightfall (434 images), normal night (500 images), night without streetlights (414 images), and rainy night (230 images) (Figure 12). Among these scenarios, the night-without-streetlights scenario involves extremely low-light conditions with minimal ambient illumination, whereas the rainy-night scenario combines low illumination with precipitation noise, presenting compound rain–night weather characteristics. The LNTU-TSD dataset contains 80 annotated images captured on highways in Northeast China (Figure 12) during heavy snowfall conditions with intense snow and accompanying light haze, presenting compound snow–haze weather characteristics. These two datasets cover three typical adverse weather conditions: extreme low-light, rain–night compound, and snow–haze compound scenarios.

Since the LNTU-TSD contains only warning and mandatory sign categories, a class mismatch with the three-category training annotations would incur evaluation penalties under the standard COCO metrics. Therefore, we report the per-category $AP_{50}$ values independently. As shown in Table 5, the CW-DETR achieves 61.8% $AP_{50}$ on warning signs, a 24.7 percentage point improvement over the RT-DETR-R18, and 90.2% $AP_{50}$ on mandatory signs, a 9.9 percentage point improvement. These results demonstrate the substantial generalization advantage of the CW-DETR for both sign categories under real-world snow conditions.

Under nightfall conditions with moderate illumination reduction, as shown in Table 6, the CW-DETR achieves a 43.8% AP, with notably higher small object detection. Under normal night conditions, the CW-DETR maintains a 2.0 percentage point advantage. In the most challenging scenario, night without streetlights, on which all of the models struggle with absolute performance, the CW-DETR still achieves a 9.0% relative improvement in terms of the AP value. Under rainy night conditions combining low illumination with precipitation noise, the CW-DETR demonstrates an 8.2% relative improvement with particularly significant gains for small objects.

**Figure 13:**
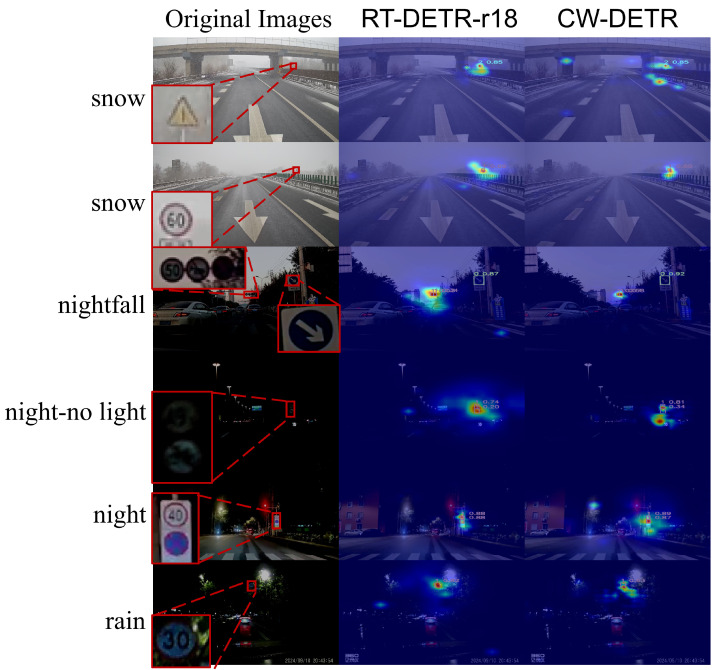
Zero-shot generalization results on the LNTU-TSD and CNTSSS datasets comparing the RT-DETR-R18 and CW-DETR. The heatmap colors range from blue (low activation) to red (high activation), indicating the intensity of model attention.

Figure 13 presents Grad-CAM visualizations across zero-shot scenarios. On the LNTU-TSD snow scenes, the CW-DETR method results in concentrated activations on traffic signs despite the snow-induced contrast reduction, whereas the RT-DETR-R18 results in more diffuse attention. Under nightfall and normal night conditions on the CNTSSS dataset, the CW-DETR achieves higher detection confidence.

However, the visualizations also reveal limitations. Under extreme conditions (night without streetlights), both models show reduced activation intensity, and the CW-DETR occasionally produces secondary activations on nontargeted bright regions such as vehicle lights. These observations indicate directions for future improvement, particularly in terms of handling extreme low-light conditions and balancing multiscale detection under compound weather interference.

## 5. Conclusions

Traffic sign detection is a fundamental perception task for intelligent transportation systems and autonomous driving, and reliable recognition under adverse weather conditions directly affects driving safety and regulatory compliance. This work proposes the CW-DETR, a real-time end-to-end framework that addresses weather-induced feature degradation through four coordinated modules: FPFENet for texture preservation, the MEEM for edge enhancement, the ADBF-FPN for cross-scale compensation, and the MCGM for noise suppression.

The experiments demonstrate that the CW-DETR achieves 69.0% AP and 94.4% ${\mathrm{AP}}_{50}$ on the CCTSDB2021 dataset, outperforming state-of-the-art detectors by 2.3–5.7 percentage points while maintaining computational efficiency (56.8 GFLOPs) suitable for vehicle-mounted deployment. Cross-dataset evaluation on the TT100K, TSRD, CNTSSS, and real-world snow conditions confirms robust generalization.

From a practical deployment perspective, the end-to-end architecture eliminates the postprocessing latency that is critical for time-sensitive driving decisions, and the demonstrated robustness across weather conditions simplifies system integration without requiring weather-specific model switching. Furthermore, our zero-shot experiments on the CNTSSS dataset’s rainy-night scenes and the LNTU-TSD dataset’s snow-haze scenes validate the adaptability of the model to two common compound weather conditions. Beyond traffic sign detection, the weather-robust feature enhancement principles demonstrated here could extend to other safety-critical tasks such as pedestrian detection, where mistake-free detection under adverse conditions is paramount, and potentially to broader domains such as wildlife monitoring where similar environmental interference challenges exist. However, real-world driving may involve more complex weather combinations or transitional lighting conditions that deserve further exploration. Additionally, variations in camera characteristics across platforms may present certain challenges for deployment. Addressing these aspects alongside the temporal consistency in video-based perception would strengthen the framework’s applicability to practical autonomous driving systems.

## Figures and Tables

**Table 1:** Ablation study: progressive integration of modules (A–D) into RT-DETR-R18 baseline.

Model	Param	GFLOPs	AP	${\mathrm{AP}}_{50}$	${\mathrm{AP}}_{75}$	${\mathrm{AP}}_{S}$	${\mathrm{AP}}_{M}$	${\mathrm{AP}}_{L}$	FPS
RT-DETR-R18	19,875,612	56.9	63.3	89.9	76.6	61.8	70.4	-	64.6
+A	13,847,100	44.5	65.5	90.9	81.0	64.4	70.6	-	104.4
+B	21,809,756	63.2	60.9	92.1	72.8	56.7	71.6	-	58.2
+C	20,108,141	57.5	62.8	90.3	74.3	62.8	64.8	-	65.4
+D	19,511,456	51.4	64.8	90.7	80.2	62.9	71.6	-	70.7
+A+B	15,900,540	52.2	64.8	92.2	79.6	64.1	68.8	-	76.8
+A+C	14,173,837	46.3	63.1	91.1	75.5	61.1	70.2	-	70.7
+A+B+C	16,473,037	54.6	65.0	91.4	76.5	62.9	72.9	-	74.6
+A+B+C+D	16,883,149	56.8	69.0	94.4	81.4	68.3	73.4	-	67.7

**Table 2:** False positive analysis: background misclassification distribution across modules.

Model	BG→0	BG→1	BG→2	${\mathrm{Acc}}_{2}$	MCA
RT-DETR-R18	0.38	0.59	0.03	0.81	89.3%
+A (FPFENet)	0.17	0.71	0.12	0.84	91.7%
+B (MEEM)	0.27	0.55	0.18	0.84	90.7%
+C (ADBF-FPN)	0.40	0.47	0.13	0.84	90.7%
+D (MCGM)	0.33	0.67	0.00	0.77	86.3%
CW-DETR	0.33	0.64	0.03	0.94	95.0%

**Table 3:** Performance comparison across real-time, end-to-end, and real-time end-to-end detectors.

Model	Param	GFLOPs	AP	${\mathrm{AP}}_{50}$	${\mathrm{AP}}_{75}$	${\mathrm{AP}}_{S}$	${\mathrm{AP}}_{M}$	${\mathrm{AP}}_{L}$	FPS
*Real-time Detectors *									
YOLOv5m	21,496,969	54.9	55.5	81.9	65.2	51.8	68.3	-	103.4
YOLOv5l	53,133,721	134.7	58.5	82.6	69.3	56.3	66.8	-	46.7
YOLOv8m	25,858,057	79.1	60.1	85.0	70.7	59.4	65.2	-	79.8
YOLOv8l	43,608,921	164.8	60.3	84.9	72.3	57.3	71.3	-	33.2
YOLOv10m	15,314,905	58.9	50.8	74.2	60.0	47.0	64.4	-	105.2
YOLOv10l	24,311,641	120	50.5	73.1	60.1	46.9	63.8	-	38.3
YOLOv11m	20,032,345	67.7	58.5	83.7	69.9	55.8	66.7	-	90.1
YOLOv11l	25,281,625	86.6	57.7	83.0	71.6	54.7	69.4	-	40.9
Hyper-YOLO	358,887	9.5	51.1	75.9	60.0	47.0	68.4	-	272.1
*End-to-end Object Detectors*									
AlignDETR	-	-	69.6	94.1	84.7	69.3	71.8	-	-
Deformable DETR	-	-	62.2	63.4	75.5	60.8	68.4	-	-
*Real-time End-to-end Object Detectors*									
RT-DETR-R18	19,875,612	56.9	63.3	89.9	76.6	61.8	70.4	-	64.6
RT-DETR-R34	31,109,315	88.8	62.5	90.9	72.4	61.2	67.9	-	45.9
RT-DETR-R50	41,960,273	129.6	65.4	92.3	83.2	64.7	69.6	-	36.6
RT-DETR-R50-m	36,257,105	96.1	64.3	92.0	76.9	63.2	70.9	-	40.3
RT-DETR-l	31,990,161	103.4	64.7	89.9	78.4	63.1	70.1	-	54.2
CW-DETR	16,883,149	56.8	69.0	94.4	81.4	68.3	73.4	-	67.7

**Table 4:** Transfer learning results on the CCTSDB2021, TT100K, and TSRD datasets.

DATA	Model	AP	${\mathrm{AP}}_{50}$	${\mathrm{AP}}_{75}$	${\mathrm{AP}}_{S}$	${\mathrm{AP}}_{M}$	${\mathrm{AP}}_{L}$
CCTSDB2021	RT-DETR-R18	63.3	89.9	76.6	61.8	70.4	-
	CW-DETR	69.0	94.4	81.4	68.3	73.4	-
TT100K	RT-DETR-R18	63.8	78.5	74.7	52.6	65.7	75.3
	CW-DETR	67.7	83.0	81.0	55.7	69.7	80.9
TSRD	RT-DETR-R18	88.0	94.4	94.2	60.5	88.2	87.8
	CW-DETR	88.4	94.7	94.7	58.6	88.6	89.0

**Table 5:** Zero-shot evaluation on LNTU-TSD real snow dataset (models trained on CCTSDB2021).

Model		${\mathrm{AP}}_{50}$
RT-DETR-R18	warning	37.1
	mandatory	80.3
CW-DETR	warning	61.8
	mandatory	90.2

**Table 6:** Zero-shot evaluation on the CNTSSS dataset under four night/rain conditions.

Model	Condition	AP	${\mathrm{AP}}_{50}$	${\mathrm{AP}}_{75}$	${\mathrm{AP}}_{S}$	${\mathrm{AP}}_{M}$	${\mathrm{AP}}_{L}$
RT-DETR-R18	nightfall	0.414	0.635	0.447	0.277	0.410	-
	night	0.351	0.572	0.389	0.246	0.424	-
	no light	0.212	0.417	0.183	0.189	0.238	-
	rain	0.282	0.454	0.299	0.192	0.323	-
CW-DETR	nightfall	0.438	0.675	0.488	0.303	0.431	-
	night	0.371	0.586	0.424	0.292	0.431	-
	no light	0.231	0.460	0.205	0.191	0.267	-
	rain	0.305	0.473	0.347	0.243	0.334	-

## Data Availability

The CCTSDB2021, TT100K, CNTSSS, and TSRD datasets are publicly available. The LNTU-TSD dataset and trained model weights are available from the corresponding author upon reasonable request.
